# Artificial Intelligence for American Society of Anesthesiologists Physical Status Classification: Agreement with Clinician Consensus and Temporal Stability Analysis

**DOI:** 10.3390/jcm15103871

**Published:** 2026-05-18

**Authors:** Anne Lykke Soerensen, Christina Froeslev-Friis, Gunhild Kjaergaard Andersen, Swati Bhavsar, Lisbeth Holmgaard Quitzau, Vinayak Desurkar, Anil Rao, Sri Krishna, Reshma Bhosale, Rajesh Prabhakar Bhavsar

**Affiliations:** 1 Department of Anaesthesia and Intensive Care, South Jutland Hospital, 6200 Aabenraa, Denmark; anne.lykke.sorensen4@rsyd.dk (A.L.S.); christina.froeslev-friis2@rsyd.dk (C.F.-F.); gunhild.kjaergaard-andersen2@rsyd.dk (G.K.A.); swati.rajesh.bhavsar@rsyd.dk (S.B.); lisbeth.holmgaard.quitzau2@rsyd.dk (L.H.Q.); 2 Department of Regional Research Unit, University of Southern Denmark, 6200 Aabenraa, Denmark; 3 Department of Cardiothoracic Anaesthesia, Deenanath Mangeshkar Hospital, Pune 411004, India; devina@hotmail.com; 4 Department of Anaesthesiology, The Royal Orthopaedic Hospital NHS Foundation Trust, Birmingham B31 2AP, UK; sufentanil@doctors.org.uk; 5 Department of Anaesthesiology and Pain Medicine, University Hospitals Birmingham NHS Foundation Trust, Birmingham B15 2WB, UK; sri.krishna@uhb.nhs.uk; 6 Department of Anaesthesiology, Doncaster Royal Infirmary, Doncaster and Bassetlaw Teaching Hospitals NHS Foundation Trust, Doncaster DN2 5LT, UK; reshma.bhosale@nhs.net

**Keywords:** American society of anesthesiologists physical status classification, artificial intelligence, anesthesiology, clinical decision-making, machine learning, natural language processing, observer variation, reproducibility of results

## Abstract

**Background:** The American Society of Anesthesiologists Physical Status (ASA-PS) classification is widely used for perioperative communication and research with known variation in agreement amongst assessors. Large language models (LLM) are increasingly considered for uniform decision making due to agreement challenges within clinicians under identical inputs. The study compared four contemporary LLMs against clinician-derived consensus and quantified within-model stability across repeated assessments. **Methods:** In a cross-sectional vignette study, 228 anesthesiologists from Denmark, England, and India classified 20 standardized perioperative vignettes through online survey. The modal response per vignette was considered as clinician consensus. Four LLMs (ChatGPT-5.2 Plus, Gemini 3 Pro, Perplexity AI Pro, Claude 4 Sonnet) received same set of vignettes through identical prompts. Temporal stability was assessed by repeating each vignette query nine times per model (three-time windows across three nonconsecutive days) in fresh sessions. Primary outcome was exact agreement with clinician consensus. **Results:** Consensus agreement for modal LLM classifications was 18/20 (90%) for ChatGPT, 17/20 (85%) for Gemini, 17/20 (85%) for Claude, and 15/20 (75%) for Perplexity. Disagreement is clustered in vignettes with weak or split clinician consensus. Stability differed by model: Claude was fully stable across all vignettes (20/20), Gemini 19/20, ChatGPT 18/20, and Perplexity 14/20; instability typically involved adjacent-class shifts. **Conclusions:** Contemporary LLMs often match clinician modal judgement and are largely temporally stable, with discordance concentrated in clinically ambiguous boundary cases.

## 1. Introduction

The American Society of Anesthesiologists Physical Status (ASA-PS) classification system is a globally used scale intended to describe preoperative health status and systemic disease burden of the patient prior to anesthesia. It categorizes patients from ASA-PS I to VI and allows an “E” modifier for emergency procedures. Although frequently interpreted as a perioperative “risk score,” ASA-PS was originally developed as a descriptive classification for standardization and reporting, and its simplicity remains both a strength and a limitation [1]. Despite its conceptual simplicity, ASA-PS has demonstrated meaningful associations with perioperative outcomes and is therefore embedded in routine pre-anesthetic assessment, perioperative communication, and observational research [2].

In contemporary practice, ASA-PS is routinely recorded in anesthesia information systems and is widely used for case-mix description and risk adjustment in registries and clinical studies [3]. In several healthcare systems, it is also applied in operational and administrative contexts, including preoperative triage pathways and, in some settings, reimbursement modifiers that assume increasing ASA-PS class reflects increasing anesthetic workload and resource utilization. This extended use implicitly requires that ASA-PS be assigned accurately, consistently and reproducibly [4].

Using ASA-PS as a proxy for patient severity to guide resource allocation has intuitive appeal because higher ASA-PS classes correlate, on average, with worse outcomes. However, ASA-PS does not explicitly incorporate procedure-specific risk, frailty, functional capacity, or surgical invasiveness. Consequently, reliance on ASA-PS for high-stakes downstream decisions such as triage level, postoperative destination, benchmarking, or administrative categorization, may be particularly vulnerable to misclassification [5]. Administrative reliance may also introduce incentive structures around scoring. Work on potential payer-linked upcoding illustrates why reproducibility matters when ASA-PS influences operational or financial decisions, even in the absence of overt systematic fraud [6].

There is extensive literature demonstrating, at best, moderate inter-rater reliability for ASA-PS in clinical practice, underscoring its dependence on clinical judgment [7]. Disagreement is most pronounced at common category boundaries (II vs. III; III vs. IV), where clinicians may differ in their interpretation of systemic disease severity, disease control, and functional limitation [8]. Variability has also been observed across provider groups and clinical contexts, including discordance between surgeons and anesthesiologists, underscoring that ASA-PS may function in practice as a clinician-dependent judgment, particularly in borderline cases [9]. Efforts to improve consistency, such as education and consultation of official definitions, can increase accuracy but do not eliminate disagreement [10].

Recent advances in machine learning and natural language processing have enabled development of models that assign or predict ASA-PS using structured electronic health record variables or pre-anesthetic text summaries, explicitly aiming to address limitations in human reproducibility [11]. At the same time, large language models based on artificial intelligence (AI) systems, have demonstrated vulnerabilities including hallucinations, instruction sensitivity, and hidden biases [12]. As these models evolve rapidly, it remains unclear whether newer iterations achieve improved agreement with clinicians, greater internal stability, or simply different patterns of inconsistency when compared with human judgment.

Accordingly, this study evaluates multiple contemporary AI models under identical conditions, comparing their ASA-PS classifications with a clinician-derived consensus and assesses the temporal stability of AI-generated assignments across repeated assessments.

## 2. Materials and Methods

This was a cross-sectional, survey-based study designed to evaluate inter-clinician variability in ASA-PS classification and to compare clinician-derived consensus with outputs from multiple contemporary LLMs. In addition, AI models were evaluated using a repeated-measures design to assess the temporal stability of ASA-PS assignments under identical input conditions.

The clinical vignettes were intentionally constructed by the author group as standardized test cases rather than as an exhaustive representation of perioperative scenarios. The objective was to enable controlled assessment of inter-clinician agreement and temporal variability in AI outputs under comparable conditions, rather than to assess performance across the full spectrum of real-world clinical complexity. The study protocol was prospectively registered on the Open Science Framework (OSF).

Twenty clinical case vignettes were developed to reflect a broad range of preoperative scenarios commonly encountered in anesthesia practice. Each vignette included sufficient demographic and clinical information to permit ASA-PS classification (I–IV, with or without emergency modifier [E]). ASA-PS I and V cases were intentionally excluded to focus on routine elective and semi-urgent perioperative assessments.

Vignettes were prepared in both English and Danish (Supplementary Material Files S1 and S2). Content equivalence between language versions was ensured through forward translation followed by independent clinical review.

Six senior anesthesiologists with more than 10 years of clinical experience (two each from Denmark, England, and India) independently reviewed all vignettes to confirm clinical realism, clarity, and sufficiency of information for ASA-PS assignment. Final vignette versions were agreed upon by consensus among the author group following iterative review. These reviewers did not contribute to outcome determination, and their assessments were not used as reference classifications.

Anesthesiologists from multiple hospitals across India, Denmark, and England were invited to participate through professional networks. Recruitment followed a pragmatic online approach using snowball sampling, whereby initial recipients were encouraged to forward the questionnaire to eligible colleagues within their professional circles.

Data collection was conducted over a predefined period from 1 November 2025 to 1 December 2025. Eligible participants who completed the survey within this period were practicing anesthesiologists involved in perioperative care. No formal exclusion criteria were applied. All responses received during this period were included in the analysis, and no formal sampling frame or random selection was applied.

Participants were drawn from multiple countries and practice settings, introducing a degree of heterogeneity in clinical background. However, detailed demographic variables such as years of experience, subspecialty, and institutional characteristics were not systematically collected and are acknowledged as limitations.

Participants completed an anonymous online survey in which they independently assigned an ASA Physical Status (ASA-PS) class (I–IV, ±E) to each of the 20 standardized clinical vignettes. Participants were blinded to AI-generated classifications and to the responses of other clinicians.

All respondents assessed the same set of vignettes, ensuring that comparisons were based on identical clinical information across participants. The use of standardized vignettes was intended to minimize variability related to local clinical environments and enhance comparability of ASA-PS assessments.

No formal a priori sample size calculation was performed. The study was designed as a pragmatic cross-sectional survey and participant inclusion was based on responses received during a predefined recruitment period. All eligible anesthesiologists who completed the survey within this period were included in the final analysis. The study aim was descriptive and comparative, focusing on agreement between ASA-PS classifications assigned to standardized vignettes rather than testing a single prespecified hypothesis.

The reference standard for analysis was defined as the modal ASA-PS classification assigned by the participating anesthesiologists (*n* = 228) for each vignette. This approach reflects collective clinical judgement rather than a definitive measure of clinical accuracy.

Response options included most relevant ASA-PS categories (I–IV), together with two additional verification options (VO): (1) request for additional information and (2) request for physical patient assessment. Selection of multiple options was permitted to reflect real-world clinical decision-making under uncertainty. This response structure was finalized following consensus discussion among the six senior anesthesiologists and was applied identically to human respondents and AI models.

For each vignette, the majority clinician responses were used to define the consensus ASA-PS classification. In cases of tied responses between ASA-PS classes, the lower class was selected to reflect conservative clinical judgment. In the event of a tie between emergency (E) and non-emergency classifications within the same class, the emergency designation was selected. The resulting clinician consensus served as the reference comparator for AI-generated classifications.

Four contemporary web-based large language model platforms were evaluated: ChatGPT-5.2 Plus, OpenAI, San Francisco, CA, USA; Gemini 3 Pro, Google LLC, Mountain View, CA, USA; Claude 4 Sonnet, Anthropic PBC, San Francisco, CA, USA; and Perplexity AI Pro, Perplexity AI, Inc., San Francisco, CA, USA. The models were accessed through their publicly available web interfaces between 16 and 21 January 2026. Because these platforms are web-based systems and exact backend version identifiers and generation parameters are not fully user-controlled, the model names displayed in the user interface at the time of access were recorded as the software/model versions.

These models were selected to represent widely accessible, general-purpose large language models from independent developers that are commonly used by clinicians in real-world settings. They differ in underlying design philosophy, safety orientation, and information retrieval strategies, allowing comparison across heterogeneous AI systems rather than variants of a single architecture. Model selection was therefore based on clinical relevance, diversity of AI approaches, and methodological feasibility rather than on theoretical performance benchmarking.

The models were accessed via publicly available web-based interfaces, where generation parameters and exact version identifiers are not user-controlled. To address potential variability, each vignette was evaluated repeatedly at predefined time points using identical prompts. This design enabled assessment of temporal stability under real-world conditions.

Each model received the same 20 vignettes using a fully standardized prompt (Table 1). The prompt instructed the model to assign at least one ASA-PS category unless classification was impossible and allowed parallel selection of VOs. Agreement proportions were calculated for each model, and 95% confidence intervals were estimated using a binomial distribution.

To evaluate temporal stability of AI-generated ASA-PS classifications, each model was queried nine times per vignette across three predefined time-of-day windows on three nonconsecutive days (3 × 3 design), using identical prompts in new, independent sessions to avoid contextual carryover. Queries were scheduled according to Copenhagen time and distributed across periods of presumed lower and higher system usage to approximate real-world conditions (Supplementary Material File S6). For each vignette and model, a modal ASA-PS class was defined as the most frequently selected classification across the nine runs. Run-level outputs, including verification option selections, were recorded, and agreement with clinician consensus was quantified per vignette and model (0–9 concordant runs). Complete run-level outputs and agreement metrics are provided in Supplementary Materials Files S3 and S4.

ASA-PS classification and VOs were treated as orthogonal outputs. ASA-PS consensus calculations and agreement analyses were based exclusively on selected ASA-PS categories, independent of any VOs. VOs were analyzed separately as markers of residual uncertainty.

**Figure 1:**
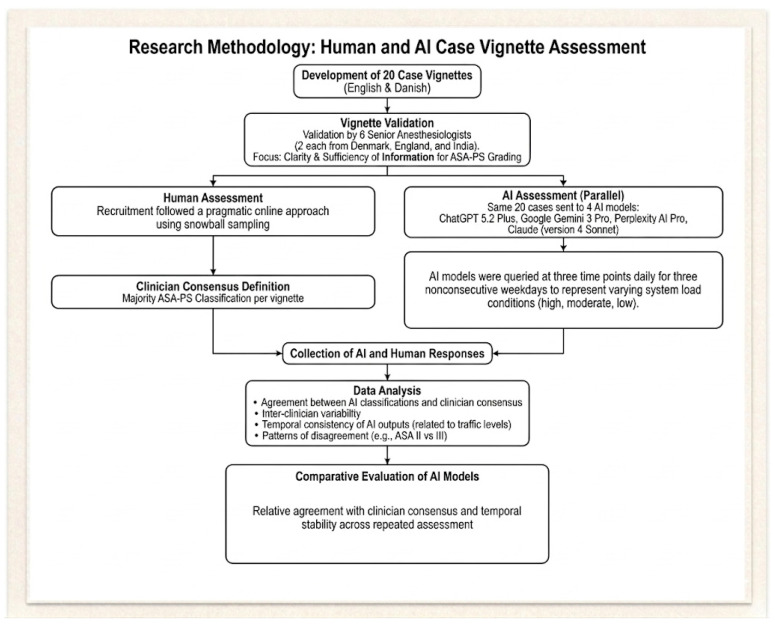
Clinical vignettes were developed and validated by senior anesthesiologists and assessed independently by practicing anesthesiologists and multiple artificial intelligence models. (Supplementary Material Files S1 and S2) Models were queried repeatedly using identical prompts across predefined time windows to evaluate temporal stability.

Primary outcomes were agreement between AI-generated ASA-PS classifications and clinician-derived consensus, expressed as percentage agreement. Secondary analyses included temporal consistency of AI responses across repeated assessments, patterns of disagreement between adjacent ASA-PS classes (e.g., II vs. III), frequency of emergency modifier assignment, and differences in agreement and stability across models. Vignettes were categorized based on the level of clinician consensus, defined pragmatically as strong (≥70% agreement), moderate (55–69%), and weak/split (<55%). These thresholds were selected for descriptive purposes within the dataset and do not represent validated or universally established cut-offs. The whole flow of study is shown in Figure 1.

Although the study was not prospectively designed using the CHERRIES checklist, key recommended elements were addressed in the design and reporting of this online survey.

## 3. Results

The analyses were structured hierarchically. Clinician-derived consensus served as the reference standard (Table 2). Intrinsic AI response behavior was characterized independently (Table 3). Agreement between AI and clinician consensus was assessed at the vignette level using modal ASA-PS classifications (Table 4). The internal reliability of AI modal classifications and their agreement with clinician consensus across repeated runs are reported in the Supplementary Materials (Supplementary Material File S4).

A total of 228 anesthesiologists completed the survey and were included in the analysis, comprising 180 non-Danish and 48 Danish respondents. All participants assessed all 20 vignettes.

For each vignette, the most frequently selected ASA-PS class constituted the clinician consensus (Table 1). VOs, defined as requests for additional information or physical examination, were analyzed separately and could co-occur with ASA-PS grading. Based on the proportion of respondents selecting the modal ASA-PS class, five vignettes (1, 4, 12, 13, and 15) demonstrated strong consensus (≥70%), eight (2, 3, 8, 9, 10, 11, 16, and 18) moderate consensus (55–69%), and seven (5, 6, 7, 17, 19, and 20) weak or split consensus (<55%). Full distributions of human responses, including VO selection, are provided in Supplementary Material File S2.

Each AI model generated an ASA-PS classification for every vignette in every run. For each vignette and model, a modal ASA-PS class was defined as the most frequently selected class across the nine repeated runs (Table 2). In many vignette–model combinations, the modal ASA-PS class was selected in all 9/9 runs, indicating fully stable internal AI consensus. In contrast, a small number of borderline vignettes showed lower internal agreement, with modal selections occurring in as few as 5/9 runs (≈55%), reflecting sensitivity to interpretive ambiguity. Complete run-level AI outputs and VO selections across all nine assessments are provided in Supplementary Material File S3. Run-level agreement with clinician consensus for each model and vignette (0–9/9 concordant runs) is provided in Supplementary Material File S4.

Exact agreement between the AI modal ASA-PS class and the clinician consensus classification across all 20 vignettes is shown in Supplementary material File S5. ChatGPT agreed with the clinician consensus in 18/20 vignettes (90%; 95% CI 68–99%), Gemini and Claude both in 17/20 vignettes (85%; 95% CI 62–97%), and Perplexity in 15/20 vignettes (75%; 95% CI 51–91%).

When stratified by clinician consensus strength, agreement was highest in vignettes with strong consensus, remained high in moderate-consensus vignettes, and decreased in weak or split-consensus scenarios (Table 4). Accordingly, AI–human discordance was largely confined to cases in which clinicians themselves demonstrated limited agreement. Confidence intervals overlapped across models, indicating that differences in agreement should be interpreted descriptively rather than as statistically significant. The overlap of confidence intervals suggests that observed differences between models may reflect sampling variability rather than true performance differences.

Across repeated assessments, AI output stability differed between models but was largely vignette-specific rather than random. Claude demonstrated complete vignette-level stability, assigning the same ASA-PS class in all nine runs for all 20 vignettes (20/20). Gemini was stable in 19/20 vignettes, ChatGPT in 18/20 vignettes, and Perplexity in 14/20 vignettes (Supplementary Material File S4). Where instability occurred, it most often reflected occasional shifts between adjacent ASA classes or parallel changes in VOs rather than large category transitions (Supplementary Material File S4). Importantly, in vignettes where AI models consistently disagreed with clinician consensus (notably vignettes 5, 10, and 20), disagreement was reproducible across all nine runs for each model, indicating stable alternative interpretations rather than stochastic variation.

## 4. Discussion

This study examined how consistently contemporary large language models assign ASA-PS classifications and how closely their outputs align with the modal judgement of practicing anesthetists when applied to standardized written vignettes. Overall, agreement between AI models and clinician consensus was high, and most AI outputs were highly reproducible across repeated assessments. Importantly, disagreement between AI and humans was not randomly distributed but concentrated in the same vignettes where clinicians themselves showed limited agreement.

A substantial body of literature has demonstrated that inter-rater reliability for ASA-PS classification in routine clinical practice is moderate at best, reflecting the system’s reliance on subjective clinical judgement. Disagreement is particularly common at boundaries between adjacent categories, most notably between ASA-PS II and III and between ASA-PS III and IV, where interpretation of systemic disease severity, disease control, and functional limitation may differ between clinicians [5].

In scenarios with strong human consensus, agreement between AI and clinicians was uniformly high across all models. Conversely, the vignettes that generated systematic AI–human discordance were those characterized by weak or split human consensus. This pattern indicates that disagreement primarily reflects intrinsic ambiguity in the vignette descriptions rather than gross model error. In other words, AI–human friction emerged precisely at points where ASA-PS classification is already challenging for clinicians [13,14].

Prior studies have similarly shown that ASA-PS classification variability increases in patients with multiple comorbidities, borderline functional capacity, or partially controlled disease, suggesting that disagreement is driven more by case complexity than by lack of familiarity with ASA-PS definitions. For example, a large German study including 684 adult general surgical patients likewise reported decreasing agreement with increasing numbers of systemic diseases. Older patient age (≥75 years) was likewise associated with lower agreement. Interestingly, agreement did not differ significantly between junior and senior anesthesiologists, indicating that ASA-PS classification need not be restricted to more experienced clinicians.

A notable finding was that both clinicians and several AI models frequently paired a definite ASA-PS classification with explicit signals of uncertainty. In weak-consensus vignettes, clinicians still assigned ASA grades but distributed them across adjacent categories, while many simultaneously indicated a need for additional information or physical examination. Gemini, ChatGPT, and Perplexity showed similar surface behavior in a subset of these borderline cases, whereas Claude consistently produced definite classifications without uncertainty modifiers.

However, the meaning of uncertainty differs fundamentally between humans and AI. For clinicians, requesting additional information or physical examination represents an actionable plan that may alter the final ASA-PS classification. For AI models, uncertainty modifiers cannot trigger further data acquisition and therefore function only as indicators that the provided text is insufficiently discriminative. In this context, AI uncertainty should be interpreted as a flag for human review rather than as a directive for a specific next step [15].

Future systems could enhance clinical usefulness by indicating which information domains—such as functional capacity, disease stability, or recent investigations—are driving the classification ambiguity.

The observed instability in a minority of vignette–model combinations is best understood as sensitivity to borderline definitions rather than random fluctuation. When a vignette clearly satisfies the textual criteria for a single ASA-PS class, all models repeatedly return the same classification. When the description lies close to a class boundary, small differences in how individual words or phrases are weighted can occasionally shift the classification to an adjacent category on repeated runs. Thus, limited instability reflects responsiveness to ambiguity in the input rather than unreliability.

Differences between models appear to reflect underlying design choices. More deterministic systems, exemplified here by Claude, commit to a single dominant interpretation and therefore achieve perfect reproducibility, even when that interpretation differs from the human modal response in some borderline cases. Other models, such as ChatGPT and Gemini, occasionally move between neighboring classes, sometimes aligning and sometimes diverging from the clinician consensus. Perplexity both disagreed more frequently and fluctuated more in ambiguous scenarios. These patterns suggest stable, model-specific interpretations of incomplete descriptions rather than erratic behavior.

The three vignettes that showed complete and reproducible disagreement across all models are particularly informative. These cases likely represent borderline ASA-PS constructs where routine clinical gestalt and literal text-based interpretation diverge in a systematic manner. Such findings highlight that clinician consensus itself does not represent an objective ground truth but rather a collective professional judgement that may incorporate tacit assumptions not explicitly present in the vignette text [2,5].

From a practical perspective, two properties are central: Concordance with typical clinician judgement across cases and reproducibility of outputs when reassessed. Perfect reproducibility alone does not guarantee agreement with clinicians, as illustrated by stable but discordant outputs in some borderline vignettes. Conversely, limited shifts between adjacent classes can sometimes increase agreement with human consensus, albeit at the expense of strict repeatability. These trade-offs are important when considering how AI systems might be used in clinical practice.

ASA-PS is increasingly used beyond its original descriptive purpose, including for case-mix adjustment, benchmarking, triage pathways, and administrative categorization. In such contexts, misclassification, or inconsistent assignment may have downstream operational or interpretive consequences, underscoring the importance of reproducibility, and transparency in ASA-PS assignment [16].

Within the scope of a vignette-based evaluation, the more reproducible models, particularly Claude, and to a large extent Chat GPT and Gemini, appear suitable as supportive tools for low-stakes applications such as education, documentation standardization, and preliminary flagging of potentially higher-risk cases. However, systematic differences in complex scenarios, frequent uncertainty signals in ambiguous cases, and sensitivity to incomplete textual descriptions indicate that fully autonomous ASA-PS assignment would be premature. AI-generated ASA-PS classifications should therefore be treated as provisional suggestions requiring human confirmation, especially in borderline or high-risk patients.

### Limitations

This study has several limitations. First, no formal a priori sample size calculation was performed, as recruitment was based on a predefined survey period. However, the inclusion of 228 anesthesiologists assessing standardized vignettes generated a substantial number of observations for comparative analyses.

Second, the ASA-PS classification lacks an objective gold standard; accordingly, the reference standard was defined as the modal classification among participating anesthesiologists, reflecting collective clinical judgement rather than a definitive measure of accuracy.

Third, the use of standardized vignettes, while enabling controlled comparisons, does not capture the full complexity of real-world clinical assessment, including dynamic patient interaction, longitudinal information, and contextual decision-making, which may limit generalizability.

Fourth, recruitment via snowball sampling and inclusion of participants from selected countries may introduce selection bias and limit representation of global practice patterns.

Fifth, the analysis focused on exact agreement and descriptive comparisons. The thresholds used to define consensus strength were pragmatic and dataset-specific, and more advanced statistical approaches (e.g., weighted agreement metrics or pairwise model comparisons) were not performed. Both factors may influence interpretation and could provide additional insight in future studies.

Sixth, the study did not provide standardized ASA-PS classification examples to participants during scoring. Prior studies have shown that the inclusion of examples can improve consistency of ASA-PS assignment; therefore, variability in clinician responses in the present study may partly reflect the absence of such guidance [17,18].

Finally, AI models were evaluated at predefined time points using fixed prompts and model behaviour may change over time with updates or differ under alternative deployment conditions; therefore, findings reflect model performance at the time of assessment.

## 5. Conclusions

In summary, contemporary AI systems can generate clinically plausible ASA-PS classifications that often align with the modal judgement of experienced anesthesiologists and demonstrate high internal reproducibility. Importantly, AI–human disagreements were concentrated in scenarios where clinicians themselves showed limited consensus. These findings suggest that such systems may serve as assistive second opinions and detectors of ambiguity but do not support their use as replacements for clinical judgement. Future research should focus on prospective, real-time comparisons between clinician and AI-generated ASA-PS classifications in routine clinical practice, incorporating dynamic patient interaction, and contextual clinical information to better reflect real-world decision-making.

## Figures and Tables

**Table 1:** Standard prompt.

You are an anesthesiologist assessing a patient for anesthesia. Based on the clinical vignette provided below, select all applicable options from the list provided. More than one option may be selected if appropriate. Do not assume additional information beyond what is explicitly stated. Do not provide explanations or free-text commentary. Respond only by listing the letter(s) corresponding to the selected option(s), separated by commas if more than one is chosen and assign at least one ASA category (I–IV, with or without emergency modifier [E]) unless classification was impossible. Clinical vignette: [Insert vignette text verbatim] Response options: A. ASA I B. ASA II C. ASA III D. ASA IV E. Requires physical examination before assigning ASA

The prompt instructed models to assign at least one ASA category (I–IV, with or without emergency modifier [E]) unless classification was impossible. Parallel verification options allowed requests for additional information or physical examination. The identical prompt was used for all models and repeated assessments to ensure comparability. ASA: American Society of Anesthesiologists.

**Table 2:** Human ASA consensus and use of verification options across 20 vignettes.

Vignette	Human Consensus	Consensus Strength	ASA II	ASA II E	ASA III	ASA III E	ASA IV	ASA IV E	Needs Info	See Patient
1	II	Strong	78.3% (179)	–	16.6% (38)	–	1.6% (4)	–	12.3% (28)	13.8% (31)
2	III	Moderate	33.1% (75)	–	57.5% (131)	–	–	–	17.9% (41)	19.2% (44)
3	III	Moderate	7.4% (17)	–	63.1% (144)	–	–	–	29.4% (67)	49.8% (114)
4	III	Strong	8.3% (19)	–	71.5% (163)	–	10.3% (23)	–	24.9% (57)	30.8% (70)
5	III	Weak/Split	2.0% (5)	–	54.2% (124)	–	35.2% (80)	–	25.0% (57)	40.9% (93)
6	II	Weak/Split	50.3% (115)	–	34.8% (79)	–	1.6% (4)	–	34.9% (80)	31.3% (71)
7	III	Weak/Split	18.7% (43)	–	48.9% (111)	–	12.3% (28)	–	46.5% (106)	28.9% (66)
8	III	Moderate	11.8% (27)	–	60.8% (139)	–	22.5% (51)	–	27.4% (62)	29.8% (68)
9	II	Moderate	66.4% (151)	–	28.1% (64)	–	1.6% (4)	–	15.0% (34)	26.4% (60)
10	IV E	Moderate	11.8% (27)	0.0% (0)	5.3% (12)	11.8% (27)	32.8% (75)	62.4% (142)	19.5% (44)	45.2% (103)
11	II	Moderate	67.5% (154)	–	27.4% (62)	–	3.2% (7)	–	25.8% (59)	26.2% (60)
12	III	Strong	13.7% (31)	–	77.9% (178)	–	3.2% (7)	–	33.4% (76)	33.9% (77)
13	III	Strong	21.4% (49)	–	74.6% (170)	–	4.6% (10)	–	14.9% (34)	24.9% (57)
14	III	Moderate	26.7% (61)	–	65.1% (148)	–	5.8% (13)	–	18.1% (41)	33.2% (76)
15	II	Strong	71.0% (162)	–	24.8% (57)	–	1.6% (4)	–	18.5% (42)	23.2% (53)
16	III	Moderate	25.5% (58)	–	60.5% (138)	–	7.4% (17)	–	26.7% (61)	40.5% (92)
17	III	Weak/Split	21.6% (49)	–	50.5% (115)	–	20.7% (47)	–	23.8% (54)	31.9% (73)
18	III	Moderate	16.1% (37)	–	57.8% (132)	–	8.9% (20)	–	37.2% (85)	47.9% (109)
19	III	Weak/Split	40.9% (93)	–	54.4% (124)	–	2.0% (5)	–	12.9% (29)	31.3% (71)
20	III E	Weak/Split	–	31.4% (72)	–	50.5% (115)	1.5% (3)	9.4% (21)	23.8% (54)	37.2% (85)

Values are percentages of all respondents (*n* = 228); numbers in parentheses indicate the corresponding number of respondents. ASA classes and verification options (“needs further information” and “see patient”) are reported separately because respondents could select an ASA class and optionally one or both verification options. Human consensus represents the most frequently selected ASA class for each vignette and was calculated from ASA classifications only, irrespective of verification selections. Consensus strength was categorized as strong (≥70%), moderate (55–69%), or weak/split (<55%). Responses labelled “E” indicate emergency surgery. A complete cross-tabulation of all response combinations is provided in Supplementary Table S1. ASA: American Society of Anesthesiologists.

**Table 3:** Modal ASA class, Output stability, and use of verification options by AI.

	Vignette	V1	V2	V3	V4	V5	V6	V7	V8	V9	V10	V11	V12	V13	V14	V15	V16	V17	V18	V19	V20
Chat GPT	Modal ASA	II	III	III	III	IV	II	III	III	II	IV E	II	III	III	III	II	III	III	III	II	III E
	Modal proportion %	100	100	100	97.2	86.1	77.8	91.7	86.1	77.8	100	100	100	100	97.2	97.2	100	86.1	100	66.7	97.2
	VO requested %	5.6	2.8	2.8	2.8	22.2	16.7	22.2	8.3	11.1	8.3	16.7	5.6	0.0	5.6	0.0	11.1	11.1	22.2	0.0	0.0
Gemini	Modal ASA	II	III	III	III	IV	II	III	III	II	IV	II	III	III	III	II	III	III	III	II	III E
	Modal proportion %	100	100	100	100	77.8	100	100	100	100	100	100	100	100	100	100	100	100	100	100	100
	VO requested %	0.0	0.0	0.0	0.0	66.7	0.0	11.1	0.0	11.1	0.0	0.0	0.0	0.0	0.0	0.0	0.0	0.0	11.1	0.0	0.0
Perplexity	Modal ASA	II	III	III	III	III	II	III	III	II	IV	II	III	III	III	II	III	III	III	II	III E
	Modal proportion %	100	100	100	88.9	55.6	55.6	66.7	100	100	100	100	100	100	88.9	88.9	100	100	100	100	100
	VO requested %	22.2	0.0	0.0	0.0	11.1	44.4	44.4	33.3	33.3	11.1	66.7	11.1	0.0	11.1	0.0	44.4	44.4	66.7	0.0	0.0
Claude	Modal ASA	II	III	III	III	IV	II	III	III	III	IV	II	III	III	III	II	III	III	III	III	III E
	Modal proportion %	100	100	100	100	100	100	100	100	100	100	100	100	100	100	100	100	100	100	100	100
	VO requested %	0.0	0.0	0.0	0.0	0.0	0.0	0.0	0.0	0.0	0.0	0.0	0.0	0.0	0.0	0.0	0.0	0.0	0.0	0.0	0.0

The modal ASA class represents the most frequently assigned ASA classification across nine repeated runs for each vignette. Modal proportion (%) indicates the percentage of runs selecting this modal ASA class. Verification option (VO) requested (%) represents the percentage of runs in which one or both verification options (“needs further information” and/or “see patient”) were selected. Full run-level outputs for all models and vignettes are provided in Supplementary Material File S2. ASA: American Society of Anesthesiologists; VO: verification option.

**Table 4:** Agreement between AI models and anesthetist consensus, stratified by consensus strength.

AI Model	Overall Agreement (*n*/20) [95%CI]	strong Consensus (*n* = 5) [95%CI]	Moderate Consensus (*n* = 8) [95%CI]	Weak/Split (*n* = 7) [95%CI]
ChatGPT	18/20 (90%) [68–99%]	5/5 (100%) [48–100%]	8/8 (100%) [63–100%]	5/7 (71%) [29–96%]
Gemini	17/20 (85%) [62–97%]	5/5 (100%) [48–100%]	7/8 (88%) [47–99%]	5/7 (71%) [29–96%]
Claude	17/20 (85%) [62–97%]	5/5 (100%) [48–100%]	8/8 (100%) [63–100%]	4/7 (57%) [18–90%]
Perplexity	15/20 (75%) [51–91%]	4/5 (80%) [28–99%]	6/8 (75%) [35–97%]	5/7 (71%) [29–96%]

Agreement represents the number of vignettes in which each artificial intelligence model matched the human consensus classification, expressed as n/total (%). Vignettes were stratified by human consensus strength, defined as strong (≥70%), moderate (55–69%), or weak/split (<55%).

## Data Availability

Relevant data has been made available through the Supplementary Material. Additional datasets (LLMs, etc.) generated and analyzed during the current study are available from the corresponding author on reasonable request.

## Supplementary Materials

The following supporting information can be downloaded at: https://www.mdpi.com/article/10.3390/jcm15103871/s1, File S1: English-language clinical vignettes used for ASA Physical Status classification. File S2: Danish-language clinical vignettes, demonstrating linguistic equivalence with the English version. File S3: Full distribution of clinician responses for all vignettes, including ASA category selection and verification option selection. File S4: Run-level AI outputs across nine repeated assessments per vignette and model, including ASA classifications, verification option selections, and run-level agreement with clinician consensus. File S5: How often AI answers matched the human consensus; File S6: Heat map of clinician and AI ASA physical status classifications across 20 clinical vignettes. Columns correspond to clinical vignettes (V1–V20); rows represent clinician consensus and AI models. Cell color indicates the modal ASA classification (ASA II–IV). Color concordance or discordance across rows reflects agreement between AI models and clinician consensus. Black dots indicate intra-model instability (classification variation across repeated queries, Days 1–3). Disagreement clusters predominantly in vignettes with weak or split human consensus, highlighting shared areas of classification ambiguity.

## Abbreviations

The following abbreviations are used in this manuscript:

ASA-PS	American Society of Anesthesiologists Physical Status
AI	Artificial Intelligence
LLM	Large Language Model
VO	Verification Option
OSF	Open Science Framework
