# Supplementary material for: Artificial Intelligence for American Society of Anesthesiologists Physical Status Classification: Agreement with Clinician Consensus and Temporal Stability Analysis
Source: J Clin Med. 2026 May 18;15(10):3871. doi: 10.3390/jcm15103871 (PMC13206816; doi:10.3390/jcm15103871)
Supplement: Supplementary file 1 [file jcm-15-03871-s001.zip › Supplementary Material File S1-ASA_AI_Questionnaire_English.pdf]

Invitation to Participate

We hereby invite you to participate in a short and engaging questionnaire that explores how closely artificial intelligence (AI) aligns with anesthesiologists' assessments when classifying patients according to the ASA Physical Status (ASA PS).

Although ASA classification is a simple tool, assessments often vary considerably between clinicians. This variation may influence resource allocation, perioperative planning, and patient safety. It is therefore both logical and desirable to develop a more uniform and reliable approach.

With the rapid development of AI chatbots, new opportunities arise for more standardized and objective assessments. However, it remains unclear how accurately AI reflects human clinical judgment and how stable its responses are over time.

In clinical practice, ASA assessment often includes a subjective element—for example, whether additional information or a physical examination might change the final classification. It will be interesting to see whether AI models respond in a similar way: do they request additional information or suggest that the patient should be physically examined?

In parallel with this questionnaire, the same 20 cases will be presented to several AI models to record their ASA classifications and reasoning. The responses from anesthesiologists and AI systems will then be compared to assess the degree of agreement, variation, and any differences in clinical interpretation.

The questionnaire consists of 20 short, realistic clinical scenarios from anesthesiology practice. In some questions, more than one answer option may be selected.

Your participation will contribute valuable knowledge about the level of agreement among anesthesiologists and how AI assessments compare with human clinical judgment—knowledge that may help shape future decision-making in anesthesiology.

Kind regards,
Anne Lykke Sørensen
Rajesh Prabhakar Bhavsar

Questions

Question 1:
A 45-year-old man is scheduled for knee arthroscopy. He has well-controlled hypertension on one medication, no organ damage, BMI 35. At preoperative assessment, BP was 158/92, but he had skipped his medication. He has recently joined a cycling club.

- ASA II
- ASA III
- ASA IV
- I need more information before making a decision.
- I would like to examine the patient physically.

Question 2:
A 62-year-old woman scheduled for thyroid surgery. She has diabetes (HbA1c 8.5%), treated with insulin, and mild kidney disease (eGFR 58). Creatinine 135 µmol/L for several years. She still cooks daily.

- ASA II
- ASA III
- ASA IV
- I need more information before making a decision.
- I would like to examine the patient physically.

Question 3:
A 70-year-old man scheduled for laparoscopic colectomy. Previous myocardial infarction 8 months ago, EF 40–45%, no angina. Can walk the equivalent of one apartment block but avoids hills. He does crossword puzzles.

- ASA II
- ASA III
- ASA IV
- I need more information before making a decision.
- I would like to examine the patient physically.

Question 4:
A 55-year-old woman scheduled for breast surgery. She has moderate COPD, requires daily inhalation therapy, becomes short of breath after climbing one flight of stairs. Last hospital admission was 2 years ago. Daily morning cough.

- ASA II
- ASA III
- ASA IV
- I need more information before making a decision.
- I would like to examine the patient physically.

Question 5:

A 75-year-old man scheduled for elective hernia repair. EF 28–30%, angina at rest controlled with nitrates. He mowed the lawn yesterday but became tired. He lives alone and does his own shopping.
• ASA II
• ASA III
• ASA IV
• I need more information before making a decision.
• I would like to examine the patient physically.

Question 6:
A 40-year-old woman scheduled for laparoscopic cholecystectomy. BMI 39, no other diseases. She snores at night but has never been evaluated for obstructive sleep apnea. She is a schoolteacher with two children.
• ASA II
• ASA III
• ASA IV
• I need more information before making a decision.
• I would like to examine the patient physically.

Question 7:
A 68-year-old man scheduled for TUR-P. He has well-controlled atrial fibrillation and is on anticoagulation therapy. Latest INR 3.5. He experiences palpitations when climbing stairs. He plays chess in a club.
• ASA II
• ASA III
• ASA IV
• I need more information before making a decision.
• I would like to examine the patient physically.

Question 8:
A 55-year-old man scheduled for AV fistula creation. He has end-stage renal disease and has been on dialysis for 3 years, otherwise stable. He occasionally skips dialysis and develops edema. He watches cricket on TV.
• ASA II
• ASA III
• ASA IV
• I need more information before making a decision.
• I would like to examine the patient physically.

Question 9:
A 28-year-old man with acute appendicitis scheduled for emergency surgery. Mild asthma, well controlled. Last used inhaler 2 years ago. Mild cold today. He is studying engineering.
• ASA II

• ASA III
• ASA IV
• I need more information before making a decision.
• I would like to examine the patient physically.

Question 10:
A 72-year-old woman with severe aortic stenosis (valve area 0.7 cm^2, EF 30%) admitted with ileus for emergency laparotomy. She walked to the waiting room but became short of breath. She uses a hearing aid.
• ASA II
• ASA III
• ASA IV
• I need more information before making a decision.
• I would like to examine the patient physically.

Question 11:
A 52-year-old woman scheduled for laparoscopic cholecystectomy. She is being treated for hypothyroidism (TSH slightly above normal). BMI 34. She walks 3 km daily but feels more tired than before. She takes vitamins.
• ASA II
• ASA III
• ASA IV
• I need more information before making a decision.
• I would like to examine the patient physically.

Question 12:
A 67-year-old retired teacher admitted for total hip arthroplasty. He has diabetes (HbA1c 9.2%), retinopathy, creatinine 145 µmol/L. He cycles short distances but becomes short of breath on hills. He drinks one glass of wine daily.
• ASA II
• ASA III
• ASA IV
• I need more information before making a decision.
• I would like to examine the patient physically.

Question 13:
A 74-year-old woman scheduled for cataract surgery. Diabetes, EF 38–40%, NYHA II. She can work in the garden for 30 minutes without becoming short of breath but avoids stairs. Mild knee osteoarthritis.
• ASA II
• ASA III
• ASA IV
• I need more information before making a decision.

• I would like to examine the patient physically.

Question 14:
A 58-year-old man scheduled for TUR-B. He has stable exertional angina, no recent myocardial infarction, EF 50%. He occasionally has heartburn. Smokes 10 cigarettes daily.
• ASA II
• ASA III
• ASA IV
• I need more information before making a decision.
• I would like to examine the patient physically.

Question 15:
A 45-year-old woman scheduled for elective hysterectomy. HIV-positive, on treatment, CD4 count 600, minimal viral load. Hb 10.5 g/dL. She has seasonal allergic rhinitis.
• ASA II
• ASA III
• ASA IV
• I need more information before making a decision.
• I would like to examine the patient physically.

Question 16:
A 72-year-old man scheduled for colon resection. He has known COPD, uses daily inhalation therapy and nocturnal oxygen. Current SpO_2 95%. He becomes short of breath when climbing stairs but can go grocery shopping without significant difficulty. Two hospital admissions in the past year. He takes vitamin supplements and watches football.
• ASA II
• ASA III
• ASA IV
• I need more information before making a decision.
• I would like to examine the patient physically.

Question 17:
A 63-year-old woman with femur fracture due to metastatic breast cancer, feels generally well. Hb 8.9 g/dL. She still cooks independently.
• ASA II
• ASA III
• ASA IV
• I need more information before making a decision.
• I would like to examine the patient physically.

Question 18:
An 80-year-old man with colon cancer scheduled for sigmoid resection. He has atrial fibrillation and is treated with apixaban. Cardiac function has not been recently evaluated,

but there is no history of heart failure or angina. He is independent in daily activities, walks daily, and does his own shopping. Family reports occasional forgetfulness, and his GP has noted early dementia.

• ASA II
• ASA III
• ASA IV
• I need more information before making a decision.
• I would like to examine the patient physically.

Question 19:
A 76-year-old woman scheduled for laparoscopic sigmoid resection. She has early Alzheimer's disease, requires help with finances but is otherwise independent in daily activities. She takes donepezil. She has mild hypertension treated with two medications. Blood tests are normal.

• ASA II
• ASA III
• ASA IV
• I need more information before making a decision.
• I would like to examine the patient physically.

Question 20:
A 60-year-old woman presents with bowel obstruction and requires emergency laparotomy. She has well-controlled hypertension treated with two medications and type 2 diabetes with HbA1c 8.0%, treated with oral agents. She reports fatigue after climbing stairs but manages her daily shopping independently. On admission, BP 135/75 mmHg, pulse 95. She also has knee osteoarthritis and wears glasses.

• ASA III
• ASA IV
• ASA III E
• ASA IV E
