# Supplementary material for: Artificial Intelligence for American Society of Anesthesiologists Physical Status Classification: Agreement with Clinician Consensus and Temporal Stability Analysis
Source: J Clin Med. 2026 May 18;15(10):3871. doi: 10.3390/jcm15103871 (PMC13206816; doi:10.3390/jcm15103871)
Supplement: Supplementary file 1 [file jcm-15-03871-s001.zip › Supplementary Material File S2- ASAAI. final vignettes.Danish.pdf]

Invitation til deltagelse

Vi inviterer dig hermed til at deltage i et kort og spændende spørgeskema, som undersøger, hvor tæt kunstig intelligens (AI) stemmer overens med anæstesiologers vurderinger ved klassificering af patienter efter ASA Physical Status (ASA PS).

Selvom ASA klassifikationen er et enkelt værktøj, varierer vurderingen ofte betydeligt mellem klinikere. Denne variation kan have betydning for ressourcefordeling, perioperativ planlægning og patientsikkerhed. Det er derfor både logisk og ønskværdigt at udvikle en mere ensartet og pålidelig tilgang.
Med den hurtige udvikling inden for AI chatbots opstår nye muligheder for en mere standardiseret og objektiv vurdering. Det er dog fortsat uklart, hvor præcist AI afspejler den menneskelige kliniske vurdering, og hvor stabilt svarene er over tid.
I klinisk praksis indeholder ASA vurderingen ofte et subjektivt element, for eksempel om yderligere oplysninger eller en fysisk undersøgelse kunne ændre den endelige vurdering. Det bliver interessant at se, om AI modeller reagerer på samme måde: efterspørger de flere informationer eller foreslår, at patienten vurderes fysisk?
Parallelt med dette spørgeskema bliver de samme 20 cases præsenteret for flere AI modeller for at registrere deres ASA klassifikationer og begrundelser.
Svarene fra anæstesiologerne og AI systemerne vil derefter blive sammenlignet for at vurdere graden af overensstemmelse, variation og eventuelle forskelle i klinisk fortolkning.
Spørgeskemaet består af 20 korte, virkelighedsnære kliniske scenarier fra anæstesiologisk praksis. I nogle spørgsmål kan der vælges mere end ét svar.
Din deltagelse vil bidrage med værdifuld viden om, hvor stor enighed der er blandt anæstesiologer, og hvordan AI's vurderinger sammenlignes med den menneskelige kliniske dømmekraft – viden, der kan være med til at forme fremtidens beslutningsgrundlag i anæstesiologien.

Med venlig hilsen
Anne Lykke Sørensen
Rajesh Prabhakar Bhavsar

Spørgsmål 1:

En 45-årig mand skal have knæartroskopi. Han har velkontrolleret hypertension på ét præparat, ingen

---

organskader, BMI 35. Ved forundersøgelse var BT 158/92, men han havde sprunget sin pille over.
Han er for nylig startet i cykelklub.

- ASA II
- ASA III
- ASA IV
- Jeg har brug for flere oplysninger før beslutning.
- Jeg vil gerne tilse patient fysisk.

Spørgsmål 2:

En 62-årig kvinde til thyroideakirurgi. Hun har diabetes, HbA1c 8,5%, i insulinbehandling, mild nyresygdom (eGFR 58). Kreatinin 135 µmol/L i flere år. Hun laver stadig mad dagligt.

- ASA II
- ASA III• ASA IV
- Jeg har brug for flere oplysninger før beslutning.
- Jeg vil gerne tilse patienten fysisk.

Spørgsmål 3:
En 70-årig mand til laparoskopisk kolektomi. Tidligere AMI for 8 mdr. siden, EF 40–45%, ingen angina.
Kan gå svarende til en boligblok men undgår bakker. Han laver krydsord.

- ASA II
- ASA III
- ASA IV
- Jeg har brug for flere oplysninger før beslutning.
- Jeg vil gerne tilse patienten fysisk.

Spørgsmål 4:
En 55-årig kvinde til mammaoperation. Hun har moderat KOL, dagligt inhalationsbehov,
Forpustet svarende til gang på én trappeopgang. Sidste indlæggelse for 2 år siden. Morgenhoste dagligt.

- ASA II
- ASA III
- ASA IV
- Jeg har brug for flere oplysninger før beslutning.
- Jeg vil gerne tilse patienten fysisk.

Spørgsmål 5:
En 75-årig mand til elektiv hernieoperation. EF 28–30%, angina i hvile kontrolleret med nitrater.
Han slog græsset i går, men blev træt. Han bor alene og handler selv ind.

- ASA II
- ASA III
- ASA IV
- Jeg har brug for flere oplysninger før beslutning.
- Jeg vil gerne tilse patienten fysisk.

Spørgsmål 6:
En 40-årig kvinde til laparoskopisk kolecystektomi. BMI 39, ingen andre sygdomme.

Hun snorker om natten, men aldrig undersøgt for obstruktiv søvn apnø. Hun er skolelærer med to børn.

- ASA II
- ASA III

- ASA IV
- Jeg har brug for flere oplysninger før beslutning.
- Jeg vil gerne tilse patienten fysisk.

Spørgsmål 7:

En 68-årig mand til TUR-P. Han har velkontrolleret atrieflimmer og er i AK-behandling.

Seneste INR 3,5. Han mærker palpitaitoner på trapper. Han spiller skak i klubben.

- ASA II
- ASA III
- ASA IV
- Jeg har brug for flere oplysninger før beslutning.
- Jeg vil gerne tilse patienten fysisk.

Spørgsmål 8:

En 55-årig mand til AV-fistel. Han har terminal nyresygdom, har været i dialyse 3 år, ellers stabil.

Han springer nogle gange dialyse over og får ødemer. Han ser cricket i TV.

- ASA II
- ASA III
- ASA IV
- Jeg har brug for flere oplysninger før beslutning.
- Jeg vil gerne tilse patienten fysisk.

Spørgsmål 9:

En 28-årig mand med akut appendicitis til akut operation. Mild astma, velkontrolleret.

Sidst brugt inhalationsmedicin for 2 år siden. I dag let forkølet. Han læser til ingeniør.

- ASA II
- ASA III
- ASA IV
- Jeg har brug for flere oplysninger før beslutning.
- Jeg vil gerne tilse patient fysisk.

Spørgsmål 10:

En 72-årig kvinde med svær aortastenose (klapareal 0,7 cm^2, EF 30%) indlægges med ileus til

akut laparotomi. Hun gik selv til venteværelset, men blev forpustet. Hun bruger høreapparat.

- ASA II
- ASA III
- ASA IV
- Jeg har brug for flere oplysninger før beslutning.
- Jeg vil gerne tilse patienten fysisk.

Spørgsmål 11:

En 52-årig kvinde til laparoskopisk kolecystektomi. Hun er i behandling for hypothyreose

(TSH lige over normal). BMI 34. Hun går 3 km dagligt men er mere træt end før. Hun tager vitaminer.

- ASA II
- ASA III
- ASA IV
- Jeg har brug for flere oplysninger før beslutning.
- Jeg vil gerne tilse patienten fysisk.

Spørgsmål 12:

En 67-årig pensioneret lærer er indlagt for hoftealloplastik. han har Diabetes (HbA1c 9,2%), retinopati,

kreatinin 145 µmol/L. Han cykler korte ture men bliver forpustet på bakker. Han drikker et glas vin dagligt.

- ASA II
- ASA III
- ASA IV
- Jeg har brug for flere oplysninger før beslutning.
- Jeg vil gerne tilse patienten fysisk.

Spørgsmål 13:

En 74-årig kvinde til kataraktkirurgi. Diabetes, EF 38–40%, NYHA II.

Hun kan arbejde i haven 30 min uden at blive forpustet, men undgår trappegang. Mild knæartrose.

- ASA II

- ASA III

- ASA IV

Jeg har brug for flere oplysninger før beslutning.

Jeg vil gerne tilse patient fysisk.

Spørgsmål 14:

En 58-årig mand til TuR-B.Har stabil angina ved anstrengelse, ingen nylig AMI, EF 50%.

Han har halsbrand af og til. Ryger 10 cigaretter dagligt.

- ASA II
- ASA III
- ASA IV
- Jeg har brug for flere oplysninger før beslutning.
- Jeg vil gerne tilse patient fysisk.

Spørgsmål 15:

En 45-årig kvinde til elektiv hysterektomi. HIV-positiv, i behandling, CD4 600, virusload minimal.

Hb 10,5 g/dL. Hun har høfeber om foråret.

- ASA II
- ASA III
- ASA IV
- Jeg har brug for flere oplysninger før beslutning.
- Jeg vil gerne tilse patienten fysisk.

Spørgsmål 16:

72-årig mand planlagt til colonresektion.
Han har kendt med KOL. Bruger dagligt inhalationsmedicin og anvender ilt til nat. Aktuelt SpO_2 95% . Han får åndenød ved trappegang, men kan gå på indkøb unden væsentlige gener. Har haft to indlæggelser det seneste år. Han tager vitamintilskud og ser fodbold.

- ASA II
- ASA III
- ASA IV
- Jeg har brug for flere oplysninger før beslutning.
- Jeg vil gerne tilse patienten fysisk.

Spørgsmål 17:

En 63-årig kvinde med femurfraktur pga. metastatisk mamma cancer, men føler sig ok. Hb 8,9 g/dl
Hun laver stadig mad selv.

- ASA II
- ASA III
- ASA IV
- Jeg har brug for flere oplysninger før beslutning.
- Jeg vil gerne tilse patienten fysisk.

Spørgsmål 18:

En 80-årig mand med colon cancer er planlagt til sigmoidresektion. Han har atrieflimren og får apixaban.

Hjertets funktion er ikke nyligt vurderet, men der er ingen anamnese med hjertesvigt eller angina.

Han er selvhjulpen i daglige aktiviteter, går ture dagligt og klarer stadig sine egne indkøb.

Familien fortæller om lejlighedsvis glemsomhed, og egen læge har noteret tidlig demens.

- ASA II
- ASA III
- ASA IV
- Jeg har brug for flere oplysninger før beslutning.
- Jeg vil gerne tilse patient fysisk.

Spørgsmål 19:

En 76-årig kvinde er planlagt til laparoskopisk sigmoidresektion.

Hun har tidlig Alzheimers sygdom, har brug for hjælp til økonomi, men er ellers selvhjulpen i daglige aktiviteter.

Hun får donepezil. Hun har let hypertension, behandlet med to tabletter. Blodprøverne er normale.

- ASA II
- ASA III
- ASA IV
- Jeg har brug for flere oplysninger før beslutning.
- Jeg vil gerne tilse patient fysisk.

Spørgsmål 20:

En 60-årig kvinde præsenterer sig med tarmslyng og skal gennemgå en akut laparotomi.

Hun har velkontrolleret hypertension, behandlet med to præparater, og type 2-diabetes med en HbA1c på

8,0 %, behandlet med tabletter. Hun fortæller, at hun bliver træt efter at have gået op ad trapper, men klarer selv sine daglige indkøb. Hendes blodtryk ved ankomst er 135/75 mmHg, puls 95. Hun har desuden knæartrose og bruger briller.

- ASA III
- ASA IV
- ASA III E
- ASA IV E
