# Supplementary material for: Artificial Intelligence for American Society of Anesthesiologists Physical Status Classification: Agreement with Clinician Consensus and Temporal Stability Analysis
Source: J Clin Med. 2026 May 18;15(10):3871. doi: 10.3390/jcm15103871 (PMC13206816; doi:10.3390/jcm15103871)
Supplement: Supplementary file 1 [file jcm-15-03871-s001.zip › Supplementary Material File S5.pdf]

Supplementary Material File S5. How often AI answers matched the human consensus.

Vignette	1	2	3	4	5	6	7	8	9	10	11	12	13	14	15	16	17	18	19	20
**CHATGPT 5.2**	100	100	100	100	0	55,6	100	44,4	88,9	0	100	100	100	100	100	100	44,4	100	33,3	0
**Cloude**	100	100	100	100	0	100	100	100	22,2	0	100	100	100	100	100	100	100	100	100	0
**Gemini**	100	100	100	100	0	100	100	100	100	0	100	100	100	100	100	100	100	100	0	0
**Perplexity**	100	100	100	100	55,6	55,6	77,8	100	100	0	100	100	100	100	100	100	100	100	0	0

Supplementary Material File S5. Consistency of Human–AI Agreement.
Consistency of agreement was defined as the proportion of repeated AI assessments (n = 9 per vignette) that matched the human consensus ASA classification. For each AI model and vignette, agreement was counted when the AI-assigned ASA class was identical to the human consensus and expressed as a percentage of total repetitions. A value of 100% indicates that the AI agreed with the human consensus in all repetitions (9/9), 55.6% indicates intermittent agreement (5/9), and 0% indicates complete and reproducible disagreement across all repetitions.
