# Supplementary material for: Artificial Intelligence for American Society of Anesthesiologists Physical Status Classification: Agreement with Clinician Consensus and Temporal Stability Analysis
Source: J Clin Med. 2026 May 18;15(10):3871. doi: 10.3390/jcm15103871 (PMC13206816; doi:10.3390/jcm15103871)
Supplement: Supplementary file 1 [file jcm-15-03871-s001.zip › Supplementary Material File S6.pdf]

Supplementary Material 6. Time periods used to represent different system load conditions for AI querying

Traffic level	UTC time	Denmark (UTC+1)	Notes
🔴 High	15:00–22:00	16:00–23:00	Slower replies, shorter/generic, occasional issues
🟠 Moderate	07:00–14:00	08:00–15:00	Generally stable, good quality
🟢 Low	00:00–06:00	01:00–07:00	Detailed, polished, well-structured

Predefined time-of-day windows used for repeated AI querying. Time intervals were selected to reflect variation in user activity across major global time zones, thereby approximating real-world usage conditions. These intervals were used as an operational proxy and do not represent direct measurement of platform-level system load. Time windows are presented in both Coordinated Universal Time (UTC) and local Danish time (UTC+1).
